# Clinicopathological and Molecular Characteristics of Pleomorphic Invasive Lobular Carcinoma

**DOI:** 10.1155/2020/8816824

**Published:** 2020-11-22

**Authors:** Jennifer M. Segar, Ritu Pandey, Kiah J. Farr, Raymond Nagle, Lauren LeBeau, Victor J. Gonzalez, Pavani Chalasani

**Affiliations:** ^1^University of Arizona Cancer Center, Tucson, Arizona 85719, USA; ^2^Department of Medicine, University of Arizona, Tucson, Arizona 85721, USA; ^3^College of Medicine, University of Arizona, Tucson, Arizona 85721, USA; ^4^Department of Pathology, University of Arizona, Tucson, Arizona 85721, USA; ^5^Department of Radiation Oncology, University of Arizona, Tucson, Arizona 85721, USA

## Abstract

Pleomorphic invasive lobular carcinoma (PILC) is a distinct morphological and biologically aggressive variant of invasive lobular carcinoma (ILC). We hypothesized that was due to de novo activation of PI3K/Akt/mTOR pathway in PILC resulting in higher proliferation rate and markers of cell cycle activation. We identified PILC and ILC tumors and tested for PI3K/Akt/mTOR pathway activation by immunohistochemistry (PTEN and pS6K1) and gene expression analysis (by Nanostring nCounter system). Proliferation index (Ki67) was elevated in 85% of PILCs compared to 20% of ILCs (*p* < 0.007). PTEN expression was high in all while pS6K1 was high in 8/9 PILCs compared to 3/9 ILCs (*p* < 0.007). Gene expression analysis shows that PILCs have overexpression of genes involved in cell cycle proliferation, cellular proliferation, DNA damage, and repair genes but no difference in PI3K/Akt/mTOR pathway genes. PILCs are a biologically distinct group of ILC, and clinicopathological characteristics suggest they would have a more clinically aggressive behavior. In addition, our results indicate that PI3k/Akt/mTOR pathway and cell cycle proliferation are activated in majority of these tumors. Further studies are needed to investigate these mechanisms as there are approved therapies available that may benefit PILCs.

## 1. Introduction

**Figure 1:**
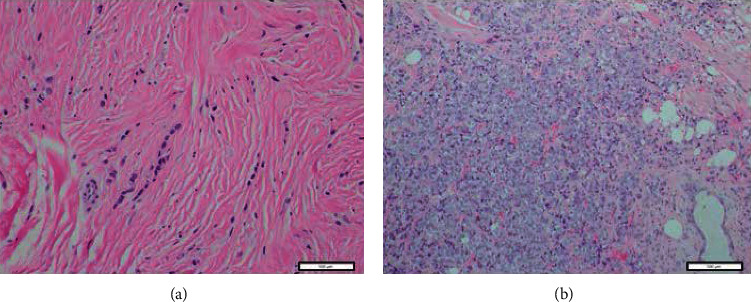
20x hemotxylin and eosin stain of (a) invasive lobular cancer and (b) pleomorphic invasive lobular cancer.

Classical invasive lobular carcinoma (ILC) is a well-established subtype of invasive breast cancer with a reported incidence of 10-15% [1]. One of the key distinguishing features of ILC is lack of E-cadherin expression noted on immunohistochemistry (IHC). Some of the reported histological subtypes of ILC are solid, alveolar, mixed, apocrine, signet-ring, histiocytoid, and tubulolobular. Pleomorphic invasive lobular carcinoma (PILC) is one category of ILC that represents approximately 15% of ILCs [2, 3]. It was first reported in 1982 by Dixon et al. who reported on 103 ILCs, some of which had nuclear pleomorphism in clusters of tumor cells [4]. Although PILC has been recognized by the World Health Organization as a distinct subtype of ILC since 2003, the clinicopathological features and prognosis of PILC are still unclear. Histologically, PILCs have more nuclear contour irregularity, increased hyperchromasia, and more frequent mitoses (Figure 1). Molecular profiling of PILC is similar to ILC in that E-cadherin expression is diminished/absent due to alterations of the *CDH1* gene on chromosome 16 [5]. Similar to ILCs, they have expression of estrogen receptor (ER) and progesterone receptor (PR); however, PILCs have been reported to have acquired further molecular alterations such as gain of HER2/neu, amplification of c-myc, and loss of p53 [3, 6, 7]. Chromosomal and array-based comparative genomic hybridization (aCGH) analysis has shown increased genomic complexity in PILC [8]. Clinically, they tend to have higher grade and stage at presentation [9]. Data regarding prognosis of PILC's is conflicting with some reporting them to be more aggressive and to have a worse prognosis compared to ILC, but others do not report such difference [9–12]. PILCs tend to have histologically more aggressive morphology suggesting they are also likely biologically distinct from ILCs. Based on the histopathological and clinical characteristics, we hypothesized that PILCs would have a higher incidence of de novo activation of phosphoinositide 3 kinase/Akt/mammalian (or mechanistic) target of rapamycin (PI3K/Akt/mTOR) pathway resulting in a higher proliferation rate and markers of cell cycle. To tests this hypothesis, we conducted a retrospective tumor tissue translational study comparing clinicopathological and molecular characteristics of PILC and ILC at our institution. To test our hypothesis, we extracted clinicopathological data, assessed expression of antigen Ki67 (marker for cell proliferation) on ILCs, and PILCs by IHC. Activation of the PI3k/Akt/mTOR pathway was evaluated by quantifying protein expression of phosphatase and tensin homolog (PTEN) and phosphorylated-S6 kinase1 (p-S6K1). PTEN is a negative regulator of the PI3K pathway, and its loss/decreased expression (by mutation or allelic imbalance) activates downstream signaling. Loss (or decrease) of the PTEN expression has been reported to be associated with PI3K pathway activation in more than 50% of estrogen receptor positive (ER+) breast tumors [13]. Since the PI3K pathway can be activated by other mechanisms in addition to PTEN loss, we hypothesized that evaluation of pS6K1 may be a better predictor of activation of this pathway compared to the PTEN protein expression alone. S6K1 is one of the best characterized downstream targets of PI3K/Akt/mTOR pathway, and its activation is regulated by various mTORC1- and PI3K-mediated phosphorylation events [14].

## 2. Materials and Methods

The University of Arizona Human Subjects Institutional Review Board approved this retrospective translational study. Our pathology database was searched to identify PILCs and ILCs from 2012 to 2014. Clinicopathological characteristics were extracted from the surgical pathology reports and medical records. Two investigators reviewed the pathology reports independently and abstracted clinocopathological data. We identified 19 cases of PILC and 126 cases of ILC from 2012 to 2014 from pathology archives at our academic institution. Initially, 20 cases of grade 1, classic ILCs were selected; however, there were technical issues with IHC staining with the tumor sections not attaching to the slide for further analysis (likely due to higher fat content). In addition, majority of those slides had minimal tumor with significant amount of fat making IHC interpretation difficult. We relooked at our database and selected 20 ILCs, which had higher tumor size, grade, and cellularity for comparison to PILCs. This was done to better match the ILC and PILC samples. Our intent for this study was to identify and match approximately 20 PILCs and 20 ILCs. Our end result is data from 20 ILCs and 19 PILCs, which is presented here. Upon further attempts to obtain samples for the project (and to stain for PTEN and S6K1), we encountered the technical issues described above regarding tumor fat content. Thus, we utilized 9 PILC and 9 ILC tissue samples to analyze the PTEN and p-S6K1 expression.

### 2.1. Immunohistochemical Analysis

Formalin-fixed, paraffin embedded archival tumor tissue was identified in 9 PILCs and 14 ILCs. Slides were reviewed by a pathologist to ensure adequate sample and quality. Routine hematoxylin and eosin (H&E) stains were performed on 3 micron sections of tissue cut from the FFPE blocks.

Immunohistochemistry (IHC) was performed for Ki67 by using Ventana Antibody #790-4286 (Rabbit primary monoclonal antibody), for PTEN by Cell Signaling Technologies #9188 (clone D4.3, XP Rabbit monoclonal antibody) and p-S6K1 by Cell Signaling Technologies, Serine 235/236 #2211. IHC was performed on a Discovery XT Automated Immunostainer (VMSI (Ventana Medical Systems), a Member of the Roche Group, Tucson, Arizona). The Ki67 expression is performed routinely at our institution on all newly diagnosed invasive breast carcinomas. It is classified as low (≤15% cells positive for expression of the antibody) or high (>15% of cells expressing Ki67). The low PTEN protein expression was defined as ≤10% of cells staining with 1+ intensity in the tumor cytoplasm or <5% of cells with 2+ intensity (i.e., cytoplasmiclongscore ≤ 10). The moderate PTEN protein expression was defined as ≥11–50% of cells staining with 1+ intensity or ≥5-25% of cells staining with 2+ intensity of their cytoplasm (i.e., cytoplasmic long score of 11-50). The high PTEN expression was defined as >50% of cells with 1+ intensity or >25% of cells with 2+ intensity. Similar quantification was applied to the p-S6K1 protein expression. IHC for PTEN and p-S6K1 was performed on 9 PILC and 9 ILC tumors.

### 2.2. RNA Extraction

Three 10 *μ* continuous unstained sections of the FFPE tumors were mounted on positively charged slides for RNA isolation. These unstained tissue slides were then incubated in a series of three baths for 2 minutes each with gentle agitation for the first 15 seconds: d-limonene (histology grade), d-limonene, and 100% ethanol. The slides were allowed to dry completely before rehydrating in a 3% glycerol (MBG) solution. A sterile razor blade was used to scrape tissue from slides for collection into a 1.7 mL microcentrifuge tube.

#### 2.2.1. RNA Isolation

RNA isolation was performed using the Roche HighPure FFPET RNA Isolation spin-column kit (Catalog #06650775001) according to manufacturer's specifications from the tumors identified on the sections. RNA was quantified using the NanoDrop 1000 (Thermo Scientific), and any with concentrations that were below 20 ng/*μ*L were concentrated using the RNA Clean and Concentrator Columns (Zymo Research, Cat #11-325) according to manufacturer's specifications.

#### 2.2.2. NanoString nCounter System Processing

100 ng of the purified RNA was hybridized with the PanCancer Code Set (Nanostring Technologies) at 65°C overnight. Further purification and binding of the hybridized probes to the optical cartridge was performed on the nCounter Prep Station, and finally, the cartridge was scanned on the nCounter Digital Analyzer. Raw counts from each gene were imported into the nSolver Analysis Software and normalized against background and housekeeping genes, and overall assay performance was assessed through evaluation of built-in positive controls.

### 2.3. Gene Expression Analysis

Log2 normalized data obtained from Nanostring nCounter analysis was analyzed for differentially expressed genes between PILC (*n* = 9) and ILC (*n* = 14). The bioinformatics analysis was carried out using R statistical tools. Limma module from Bioconductor and *t*-statistic was used for analysis of variance to estimate differential levels of transcripts. Gene alterations with significant *p* value of ≤ 0.05 were chosen as changing between the two groups. This provided a list of 28 genes. The genes were clustered, and a heat map was generated using gplots package (Figure 1—heat map showing cluster of genes associated with PILC subtype that are differentially expressed than ILC). Further correlation analysis was done for genes showing greater expression in PILC to find more genes that show similar expression trend between PILC and ILC.

## 3. Results

### 3.1. Clinicopathological Characteristics

Data was extracted from medical records and pathology reports on 19 PILCs and 20 ILCs.  Table 1 summarizes the histological data. The HER2 expression was negative in all tumors (PILCs and ILCs). In the PILC group, lymph nodes were involved with metastatic carcinoma in 37% cases (negative in 47% and unknown in 16%) whereas in the ILC group, 70% had metastatic tumor to lymph nodes (negative in 30%). The 21-gene recurrence score assay (Oncotype Dx) was performed on 10 PILCs and 6 ILCs. The 21-gene recurrence score assay has been clinically validated and is used to classify tumors into low (<18), intermediate (18-30), and high (≥31) risk groups [15]. Results demonstrated that PILCs have higher scores (median 23, range 6-36) with the majority being in the intermediate or high range (8/10). In contrast, 4/6 ILCs were in the low risk category (2 were in the intermediate range with scores of 20 and 22).

### 3.2. IHC Results

**Figure 2:**
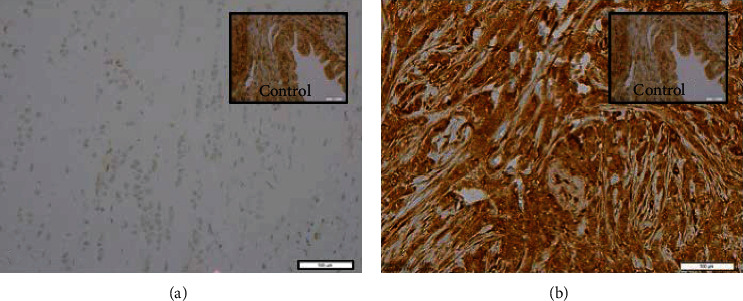
PTEN IHC results (20x for both rumors, 40x for controls): (a) PTEN negative ILC tumor with positive control; (b) high PTEN expression in a representative PILC tumor tissue with positive control.

**Figure 3:**
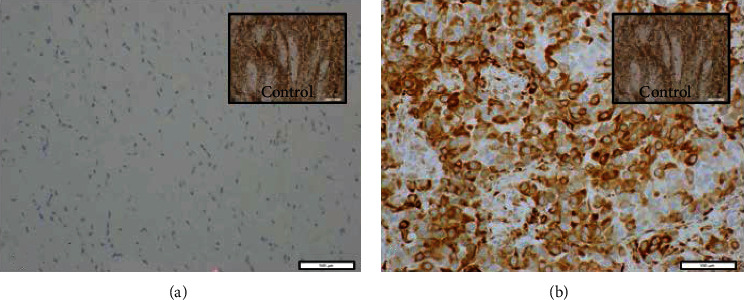
p-S6K1 IHC results (20x for both tumors, 10x for controls): (a) p-S6K1 negative ILC tumor with positive control; (b) p-S6K1 positive PILC tumor tissue with positive control.

Testing for Ki67 was performed on 19 PILCs and 20 ILCs. The proportion of tumors with ≥15% Ki67 was significantly higher in PILC group (*p* = <0.05) compared to the ILC group (Table 1). Nine PILC and 9 ILC tumors were stained for the PTEN and pS6K-1 expression. The results show high expression of PTEN in all PILC and ILC tumors (Figure 2); however, pS6K1 was high in 8/9 PILC tumors compared to 3/9 ILC tumors (*p* < 0.007) (Figure 3). Our results indicate that PI3k/Akt/mTOR pathway is activated de novo in the majority of PILCs compared to ILCs. In addition, they also suggest that PTEN is not the key regulator of PI3k/Akt/mTOR pathway as it has high expression in all the tumors.

### 3.3. Gene Expression Results

**Figure 4:**
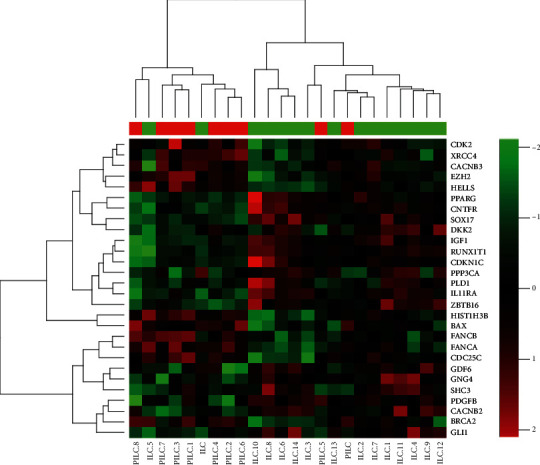
Heat map showing cluster of genes associated with PILC subtype that are differentially expressed than ILC subtype.

The heat map (Figure 4) shows clustering of genes and samples. PILC subtype does show overexpression of genes involved in cell cycle proliferation (CDC25C,CDK2), cellular proliferation (HELLS) [15], and DNA damage and repair (XRCC4, FANCA, FANCB, BRCA2) [16] genes compared to ILC. Correlation analysis of these overexpressed genes with other genes found more DNA repair and cell cycle genes that had greater expression in PILC (data not shown). We did not find any significant difference in expression of genes involved in the PI3K/Akt/mTOR pathway between the two groups (201 genes in this pathway were tested including but not limited to PTEN, PIK3, mTOR, AKT, etc.; however, S6K1 was not in these genes). Correlation analysis of these overexpressed genes with other genes found more DNA repair and cell cycle genes that had greater expression in PILC (data not shown), but they were not statistically significantly different compared to ILC.

## 4. Discussion

PILCs are a unique histological subtype of ILCs. They tend to have higher grade, more nuclear pleomorphism, single nucleolus, and increased proliferation compared to classic ILCs. They also seem to be molecularly divergent with increased proportion of tumors overexpressing HER2/neu and c-myc and having loss of p53 which are not typically seen in ILCs. Based on IHC assessment, the majority of classical ILCs tend to fall in the luminal A subtype (grade I, ER+/PR+/HER2–ve with a low Ki67). However, PILCs tend to have more of a luminal B IHC picture (grades I-III, ER+/PR+/-/HER2-/+, Ki67-high). Clinicopathological characteristics suggest that they would have a more clinically aggressive behavior, but studies in the literature have conflicting results with regard to prognosis. Given the unique histology, we sought to further assess and characterize some of the molecular features which could be distinct between the two groups. We evaluated the tumors for expression of markers for cell proliferation, cell cycle, and PI3K/Akt/mTOR pathway activation.

Activation of PI3K/Akt/mTOR pathway has been associated with endocrine and cytotoxic therapy resistance in breast cancer with the majority of them occurring in hormone receptor (HR) positive tumors [16]. There is preclinical and clinical evidence supporting that the activation of this pathway is involved in resistance to endocrine therapy in HR-positive breast tumors [17]. Endocrine resistance by this pathway activation is likely mediated through multiple mechanisms including stimulation of proliferation or survival pathway or downregulation and loss of HRs. It is now known that PIK3CA is mutationally activated in up to 40% of ER alpha-positive tumors; PTEN levels are decreased in a similar proportion, and AKT2 (up to 5%) and p70S6K (10-20%) may also be overexpressed by amplification in some breast tumors [13]. The ER-positive tumors were found to have the highest frequency of mutations in the catalytic subunit of PI3K (P13KCA); however, the presence of PIK3CA mutations in ER-positive tumors was not associated with increased phosphorylation of Akt, pS6K, and 4EBP1, which are markers of activation of the PI3K pathway [13]. Moreover, these mutations have not correlated with worse prognosis in endocrine-treated breast cancer [13, 18]. On the other hand, PTEN, a negative regulator of the PI3K pathway, activates downstream Akt/mTOR signaling and may contribute to endocrine resistance [19]. Cowden's syndrome, a result of germ-line mutation in PTEN gene, has a predisposition of breast and other cancers [20]. Sporadic PTEN mutations are rare but have been reported in breast cancer [21, 22]. Even more commonly, there is an allelic imbalance of PTEN, and the PTEN protein is absent or decreased in higher number of breast cancers. Typically lost in ER-negative breast cancer, PTEN protein is decreased in more than 50% of ER+ breast cancer [19]. Subtle downregulation of PTEN by only 20% has been shown to increase tumor invasive potential in mouse models [23]. This pathway can be activated by other mechanisms in addition to PTEN loss (like growth factor signaling, mutations, and tumor microenvironment). Currently, there are no established standard biomarkers to detect activation of this pathway. PI3K/Akt activation pathway gene signatures have been reported to predict response, but none are clinically validated for treatment decision making [18]. We evaluated expression of PTEN and pS6K as markers of this pathway activation in our study in an attempt to study protein expression as a biomarker for this pathway.

Our results indicated PILCs are clinicopathologically more aggressive than ILCs with similar phenotypes. They tend to have higher Ki67 and higher 21-gene assay recurrence score. In addition to prognosis, the 21-gene recurrence score assay is also used clinically to predict benefit from adjuvant chemotherapy. Our results indicated that chemotherapy might be beneficial in PILCs. This is in contrast to the common thinking that ILCs are not chemosensitive. Despite small numbers of PILCs tested by this assay (10), only 2 tumors were in the low range (<18). Also, the 21-gene recurrence score assay was performed on 6 ILCs which had a higher grade (similar to PILCs); however, the majority of them (4/6) were in the low range. These results suggest that biologically PILCs are likely more aggressive than ILCs.

One of the signaling pathway mechanisms known to be activated in biologically aggressive tumors is cell proliferation, cell cycling, and PI3K/Akt/mTOR pathway. We sought out to test for these pathways by IHC and RNA expression analysis.

IHC staining and comparison of Ki67 does confirm our hypothesis that there is more activation of proliferation pathways in the PILCs compared to ILCs. We also noted that PI3K/Akt/mTOR pathway is activated de novo in PILC subgroup compared to ILCs as demonstrated by increased expression of pS6K1 in PILC tumors. Further, we found that PTEN is not the primary regulator of the pathway in our sample set. Other activation mechanisms seem to play a role in these tumors and need to be investigated further. We sought to analyze other causes of activation by looking at gene expression analysis of activators of PI3K/Akt/mTOR pathway but were unable to determine a significant difference between the groups. One of the major reasons is the sample size, and the number of genes analyzed (770 genes in the Nanostring nCounter analysis) is relatively small compared to published gene expression analysis. In addition, it is important to remember that PILC is a subgroup of ILC, and an overlap of certain genes/pathways is expected. Also, attempting to find a distinction by analyzing a small sample set is difficult, and all results (positive or negative) have to be interpreted with caution.

## 5. Conclusions

PILCs are a clinically and molecularly distinct subtype of ILCs. It is important for clinicians to recognize this subtype and consider additional testing (such as the 21-gene recurrence score or other prognostic/predictive assays) to help with treatment decisions (including adjuvant chemotherapy). Given our and other results that PILCs are molecularly distinct, further studies to investigate these mechanisms appear warranted. Currently, there are medications approved which target some of these activated pathways (CDK4/6 inhibitors and mTOR inhibitors), and it would be beneficial to know which tumors might respond better to such treatments and if these treatments might be beneficial in this subgroup early on (stages I-III in the neoadjuvant or adjuvant setting) given the molecularly distinct mechanism.

## Figures and Tables

**Table 1:** Histological characteristics of PILC and ILC tumors.

Characteristic	PILC (*n* = 19)	ILC (*n* = 20)
ER+	19	20
PR+	10	13
HER2 IHC		
0	8	10
1+	7	10
2+	4	0
Grade		
1	0	6
2	10	14
3	9	0
Lymph node metastasis	7	14
Ki67 expression		
Range	5-70%	1-20%
Median	25%	10%
≥15% (high expression)	16	4
21-gene recurrence score	*n* = 10	*n* = 6
Intermediate/high	8	2

PILC: pleomorphic invasive lobular carcinoma; ILC: invasive lobular carcinoma; ER+: estrogen receptor positive; PR+: progesterone receptor positive.

## Data Availability

Data is available on request (Nanostring data and scoring sheets for IHC analysis). Please contact the corresponding author (Pavani Chalasani, MD).
